# P-1981. Sex-Based Differences in Adverse Events Associated with Common Antibiotics: A 14-Year Comparative Signal Analysis from the FAERS Database

**DOI:** 10.1093/ofid/ofaf695.2148

**Published:** 2026-01-11

**Authors:** Manu Mathew, Ashin Siby, Jose T John

**Affiliations:** Durdans Hospital, Colombo, Western Province, Sri Lanka; Durdans Hospital, Colombo, Western Province, Sri Lanka; Durdans Hospital, Colombo, Western Province, Sri Lanka

## Abstract

**Background:**

Sex-based differences in drug safety profiles are increasingly recognized as a critical component of precision medicine. Antibiotic-related adverse events (AEs) have historically lacked sex-specific analysis, despite differences in pharmacokinetics, immune response, and drug metabolism. This study investigates sex-based disproportionality in reported AEs for commonly prescribed antibiotics using the U.S. FDA Adverse Event Reporting System (FAERS) over a 14-year period.

**Figure:**
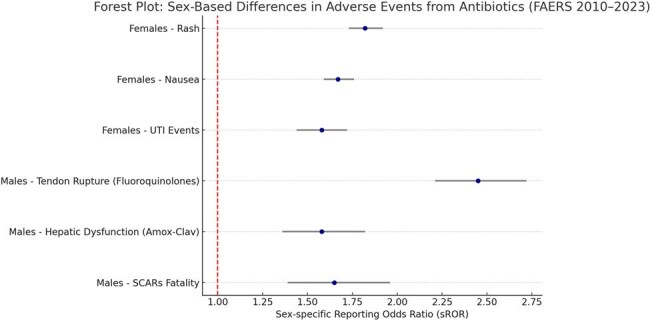
Forest Plot: Sex-Based Differences in Adverse Events from Antibiotics This forest plot presents sex-specific Reporting Odds Ratios (sRORs) with 95% confidence intervals for adverse events linked to antibiotics. Females reported higher disproportionality for rash, nausea, and urinary tract events, while males showed stronger signals for tendon rupture (fluoroquinolones), hepatic dysfunction (amoxicillin-clavulanate), and fatal SCARs. These findings underscore the clinical relevance of sex-specific risk profiling in antibiotic safety.

**Methods:**

A retrospective analysis was conducted using FAERS data from January 2010 to December 2023. Reports involving 10 widely used antibiotics (e.g., amoxicillin-clavulanate, ciprofloxacin, azithromycin, levofloxacin, doxycycline, nitrofurantoin) were included. Adverse events were stratified by sex and grouped by system organ class (SOC). Disproportionality was assessed using sex-specific Reporting Odds Ratios (sROR) and 95% confidence intervals (CI). A signal was considered significant if the lower CI >1 and ≥3 reports per event.

**Results:**

Out of 282,415 total antibiotic-related reports, 62.8% were submitted for females and 37.2% for males. Females had significantly higher signals for rash (sROR: 1.82, CI: 1.73–1.92), nausea (sROR: 1.67), and urinary tract events, particularly with nitrofurantoin. In contrast, males demonstrated higher disproportionality for tendon rupture with fluoroquinolones (sROR: 2.45, CI: 2.21–2.72) and hepatic dysfunction with amoxicillin-clavulanate (sROR: 1.58). Notably, severe cutaneous adverse reactions (SCARs) were more commonly reported by females but had higher fatality rates in males (13.5% vs. 8.2%).

**Conclusion:**

This FAERS-based analysis reveals distinct sex-based adverse event profiles for common antibiotics. These findings advocate for greater inclusion of sex-stratified data in prescribing guidelines and post-marketing surveillance. Incorporating sex as a variable in antimicrobial stewardship can lead to safer, more individualized care.

**Disclosures:**

All Authors: No reported disclosures

